# From Tradition to Innovation: The Future of Aging in Korean and Korean American Communities

**DOI:** 10.1093/geroni/igaf122.1451

**Published:** 2025-12-31

**Authors:** Kyeongmo Kim, Giyeon Kim, Nan Sook Park

## Abstract

As aging populations grow in both Korea and Korean American communities, the intersection of tradition and innovation plays an important role in shaping the health and well-being of older adults. This interdisciplinary symposium highlights research in Korean and Korean American communities, bridging traditional practices and modern innovations to address health behaviors, mental health, digital inclusion, and novel interventions. The main objectives of this symposium are to (1) examine how traditional values and emerging innovations influence aging experiences among Korean and Korean American older adults, (2) identify key social and technological determinants of well-being, and (3) discuss policy and intervention strategies that can support older adults in these communities. This session features experts in gerontology, social work, and public policy to offer a nuanced understanding of aging in Korean and Korean American communities. Dr. Yi examines Korean American older adults’ reliance on traditional complementary and alternative medicine (TCAM) due to healthcare access barriers, highlighting individual and geographic influences. Dr. Lim-Soh investigates the impact of post-retirement workforce re-entry on depressive symptoms in Korea, revealing mental health benefits of bridge employment. Dr. Kang analyzes digital skills trajectories among middle-aged and older Koreans, identifying key social and demographic determinants influencing adaptation. Finally, Dr. Kwon assesses the feasibility of a virtual reality-based psychological intervention for older Korean immigrants in the U.S., addressing mental health challenges linked to racial discrimination and the COVID-19 pandemic. By bridging tradition and innovation, this discussion provides insights into culturally responsive policies and interventions that support aging populations across borders. Korean and Korean American and Aging Interest Group Sponsored Symposium

